# Administering an eye anaesthetic: principles, techniques, and complications

**Published:** 2008-03

**Authors:** Ahmed Fahmi, Richard Bowman

**Affiliations:** Paediatric Ophthalmology Fellow, CCBRT Disability Hospital, Tanzania. Email: biophku@yahoo.com; Ophthalmologist, CCBRT Disability Hospital, Tanzania; Honorary Senior Lecturer, London School of Hygiene and Tropical Medicine. Email: richardbowman@raha.com

**Figure:**
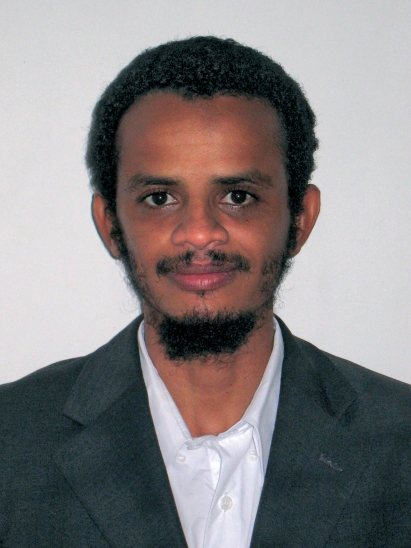


**Figure:**
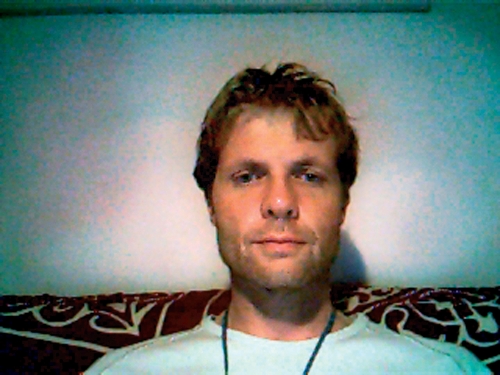


## Rationale

The trigeminal nerve carries the sensory innervation of the eye and adnexa in three divisions: ophthalmic, maxillary, and mandibular. The sensory fibres of the eye and adnexa are found in the ophthalmic division – with the exception of a portion of the sensory input from the lower lid, which is carried by the maxillary division. Blocking the sensory fibres provides **anaesthesia** so that no pain is felt.

The motor supply of the extraocular muscles and levator palpebrae superioris is carried by the oculomotor (III), trochlear (IV), and abducens (VI) nerves. Paralysing these muscles by blocking their motor supply provides **akinesia** so that the eye does not move during surgery.

The motor supply of the orbicularis oculi, which is responsible for the gentle and forcible closure of the eye, is carried by the facial nerve (VII). Blocking these fibres will provide better surgical exposure. It also reduces the risk of forcing out the ocular contents if the patient tries to close his eyelids forcibly after the surgeon opens the globe.

## Anatomy

It is important to recall the anatomy and to have a precise knowledge of the various injection sites for the anaesthetic. The anteroposterior diameter of the globe averages 24.15 mm (range: 21.7 to 28.75 mm). The axial length of myopic eyes are at the upper end of this range. This increases the risk of globe perforation, especially with a retrobulbar block. The length of the bony orbit is about 40 to 45 mm. On average, the anatomic equator is about 13 to 14 mm behind the limbus along the surface of the globe. At its closest distance to the bony orbit, the globe is about 4 mm from the roof, 4.5 mm from the lateral wall, 6.5 mm from the medial wall, and 6.8 mm from the floor.

The **retrobulbar space** lies inside the extraocular muscle cone, behind the globe. Relatively avascular areas of the orbit are confined to the anterior orbit in the lower outer (inferotemporal) and upper outer (superotemporal) quadrants. **The superonasal quadrant** is highly vascular and has limited space.

Tenon's capsule is the anterior extension of the visceral layer of dura investing the optic nerve. Therefore, the **sub-Tenon's space** is continuous with the subdural space and is, in effect, an anatomical pathway from the limbus to the retrobulbar space. Because the conjunctiva fuses with Tenon's capsule 2 to 3 mm behind the limbus, the sub-Tenon's space can be accessed easily through a scissor snip made there.

## Choosing the anaesthesia technique

Decide in advance what technique you are going to use. A **retrobulbar block** is more efficient in producing anaesthesia and akinesia and has a faster onset of action. However, it carries a higher risk of rare, yet serious, complications, such as globe perforation, retrobulbar haemorrhage, and injection of the anaesthetic into the cerebrospinal fluid (CSF). Mastering the technique reduces these risks significantly.

The probability of complications is reduced in a **peribulbar block**; however, this technique is slower and less efficient, it carries a higher risk of potential chemosis, and it puts more pressure on the eye. A retrobulbar block should be avoided if the axial length of the eye is greater than 27 mm.

When a retrobulbar or peribulbar block is unsatisfactory, you can add a **sub-Tenon's block**; it is a suitable supplement. By itself, the sub-Tenon's block is useful for shorter procedures, provided you are operating on cooperative patients. The sub-Tenon's block is more likely to be performed by an ophthalmic surgeon than by an ophthalmic anaesthetist. It enables top-up injections to be easily and safely given. Sub-Tenon's blocks are less likely to cause systemic complications than retrobulbar or peribulbar blocks.^1^

## The anaesthetic solution

### Components

**Lignocaine 2%** is the most popular agent for nerve blocks. It has a rapid onset of action and its effect will usually last for an hour. **Bupivacaine 0.5%** lasts for three hours or even longer; this anaesthetic can be useful for prolonged procedures such as vitreoretinal surgery.

**Hyaluronidase** may increase the effectiveness of a block by facilitating the spread of lignocaine or bupivacaine through the tissues. Hyaluronidase can be used in a concentration of approximately 50 units/ml (range: 25 to 75 ml).

**Adrenaline** slows the absorption of anaesthetic agents into the systemic circulation. This will provide a longer duration of action and reduce the risk of systemic toxic effects. It is used in a concentration of 1:100,000.

### Preparing the solution

- To use hyaluronidase, add one ampoule (containing 1,500 units) to a 20 ml or 50 ml bottle of lignocaine 2% or bupivacaine 0.5% (this only stays active for few days after mixing).
- To use adrenaline, add 0.1 ml from a vial of 1:1,000 adrenaline to 10 ml of the anaesthetic solution (to get 1:100,000).

**Note:** Lignocaine often comes already premixed with 1:100,000 adrenaline.

## Basic steps: all techniques

- Introduce yourself to the patient. Explain the procedure in a short, simple, and understandable way and reassure the patient.
- Check the patient's full name, the eye assigned for surgery, and the type of surgery required.
- Record blood pressure, pulse rate, and respiratory rate as a baseline for the monitoring of vital signs. These observations should be repeated every two to three minutes after the injection. Where available, pulse oximetry should be used during injection and surgery.
- Check that resuscitation equipment and medication is available to deal with a systemic complication, should one occur.
- Lie the patient flat in a safe and comfortable way, with head supported.
- Ask the patient to look straight ahead (not upwards or nasally); hold the patient's hand in front of his or her eye and ask him or her to look at it.
- Withdraw the plunger of the needle slightly before injecting the anaesthetic to make sure that you have not entered a blood vessel (blood) or the dural sheaths (CSF).
- Assess the efficiency of the anaesthesia by asking the patient to look in the four cardinal positions of gaze.
- Recall the possible complications (see box below), look out for their clinical manifestations, and be prepared to manage them. Anticipate complications in eyes with high axial length and in uncooperative patients. Staff should attend resuscitation courses annually to familiarise themselves with the handling of serious complications.

## Retrobulbar block

- Prepare the injection: 2 to 3.5 ml of the anaesthetic solution in a syringe with a sharp 23-gauge 24 mm needle (not 38 mm). The needle should not have an acute bevel.
- Feel the lower orbital rim and pass the needle through the skin or the conjunctiva at the junction of its lateral (outer) and middle thirds. The bevel of the needle should be pointing upwards. The needle should be passed straight back below the eye for 15 mm; it should be parallel to the floor of the orbit and angled down (Figure 1). You might feel the resistance as you pass through the orbital septum.
  **Figure 1:**
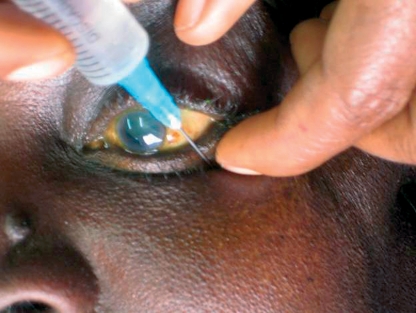
***Retrobulbar injection: the needle is passed through the junction of the middle and outer third of the inferior orbital rim, then straight back below the eye for 15 mm. The needle should be parallel to the floor of the orbit and angled down.***
- Change the direction of the needle so that the tip is pointing upwards and inwards towards the back of the skull. Feel the resistance as the needle passes through the muscle cone. The needle should be advanced not more than 24 mm from the skin in total (Figure 2).
  **Figure 2:**
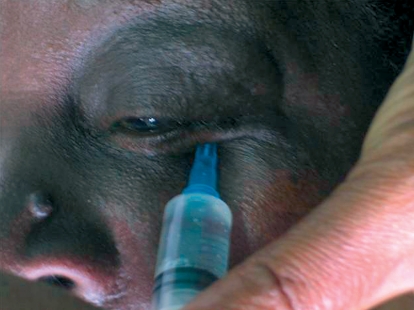
***Retrobulbar injection: the needle is advanced and the antibiotic injected after the needle's direction was changed so that the tip points upwards and inwards towards the opposite occipital eminence (slightly to the opposite side of the midline of the back of the skull). Note the drooping of the upper lid.***
- Inject slowly and look for dilation of the pupil and drooping of the upper lid.
- Close the eyelids gently, cover with a pad, and immediately apply firm, gentle pressure for 5 to 10 minutes. This can be done manually or with a special balloon inflated to 30 mmHg.

**Note:** A failed block can be repeated only once.

**Figure 4:**
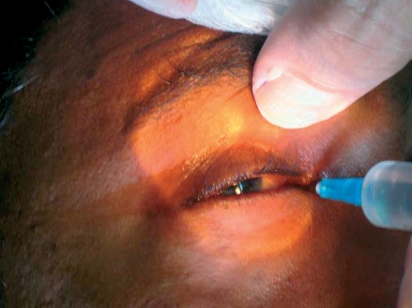
***Peribulbar block: the needle is advanced between the caruncle and the medial canthus in a backwards and medial direction, away from the globe.***

**Figure 5:**
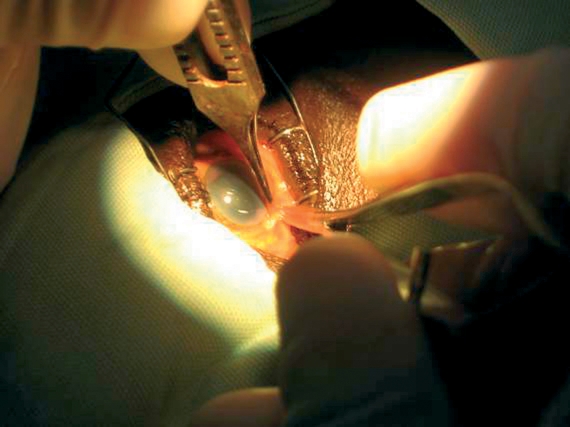
***Sub-Tenon's block: a pair of spring scissors is held perpendicularly to make a small (0.5 mm) snip incision through both the conjunctiva and Tenon's capsule, 2 to 3 mm behind the limbus in the inferomedial quadrant.***

## Peribulbar (periconal) block

This block consists of two injections; it is injected inferotemporally and between the caruncle and medial canthus.

- Prepare the syringe: 7 to 10 ml of the anaesthetic solution in the same syringe as for a retrobulbar block.
- Expose the lower fornix by pulling the lower lid down gently (Figure 3).
  **Figure 3:**
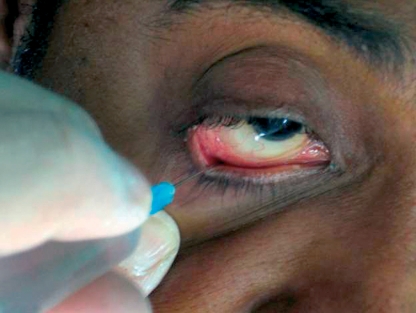
***Peribulbar block: the needle is inserted through the fornix below the lateral limbus after the lower fornix was exposed (by pulling the lower lid down gently).***
- Instil one drop of topical anaesthetic eye drops.
- Insert the needle through the fornix below the lateral limbus. Pass it backwards and laterally for not more than 24 mm. Always keep it away from the globe by directing it slightly downwards (Figure 3).
- Inject at the level of the equator
- The inferotemporal anaesthetic injection 4 to 5 ml in total) may be divided into three shots: 1 ml immediately posterior to the orbicularis oculi, 1 ml just anterior to the equator, and 2 ml after the needle is advanced past the globe.
- The second injection (3 to 4 ml in total) can be given between the caruncle and the medial canthus, then passed back and slightly medially (away from the globe) for about 24 mm, to inject 3 to 4 ml of the anaesthetic (Figure 4). Injecting directly through the caruncle can cause significant bleeding.
- The superonasal approach is now regarded as unduly dangerous and it should be abandoned. This is due to high vascularity and limited space.

## Sub-Tenon's block

- Prepare 2 to 3.5 ml of anaesthetic solution.
- Instil one drop of anaesthetic eye drops. A small swab soaked in topical amethocaine and left in the lower fornix for a minute is particularly effective.
- Cauterising the space before incision is extremely helpful in limiting both the risk of subconjunctival haemorrhage and that of an unintended extension of the incision. To do this, gently apply the bipolar cautery, barely touching but not pressing down on the conjunctival surface. This also helps to lift the Tenon's capsule away from the sclera.
- Use a pair of spring scissors to make a small (0.5 mm long) snip through both the conjunctiva and Tenon's capsule, 2 to 3 mm behind the limbus in the inferomedial quadrant of the globe. The scissors should not be opened more than halfway. It is essential to find the sub-Tenon's plane, i.e. to dissect down to bare sclera. It helps to hold the scissor blades so that their plane is perpendicular to the ocular surface instead of being parallel to it (Figure 5).
- Use a specially designed blunt cannula to inject the anaesthetic. However, if you do not have a specially designed cannula, a lacrimal cannula is a suitable alternative. Mount the cannula on a syringe containing the anaesthetic solution.
- Pass the cannula through the snip incision. The incision should fit tightly around the cannula.
- Advance the cannula backwards with its tip touching and following the curvature of the globe all the way to the retrobulbar space. As the equator is passed, the hand and syringe need to rotate away from the globe so that the cannula tip stays in the space (Figure 6). Inject the anaesthetic carefully.
  **Figure 6:**
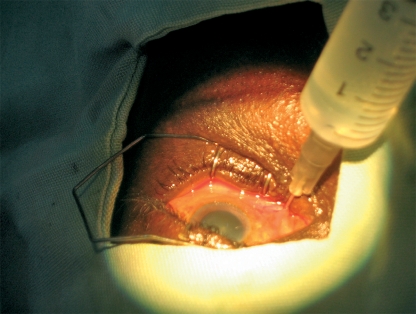
***Sub-Tenon's block: the hand and syringe is rotated away from the globe as the equator is passed so that the cannula tip stays in the space.***

**Complications of retro- or peribulbar anaesthesia**

**Retrobulbar haemorrhage** is indicated by a hard and tense orbit with no retropulsion of the globe, proptosis, and subconjunctival haemorrhage. Management is usually conservative: surgery needs to be postponed. However, if the eye is very hard, you should perform an emergency lateral canthotomy to relieve pressure on the globe: clamp the lateral canthus with an artery forceps for 30 seconds, then cut it with sharp scissors.

**Globe perforation** is a rare and serious complication. Its adverse effects can be reduced if the anaesthetic is not injected because the complication has been recognised in time. You should suspect a globe perforation if the eye becomes soft as you insert the needle. If the globe has been engaged by the needle, it will not move as you ask the patient to move his eye from side to side. Be very careful with your technique: advance the needle gently and take particular care in eyes with a high axial length (the needle should be kept further away from the globe).

**Systemic complications** are very rare but very serious when they occur – they might be fatal. These complications occur if the local anaesthetic was injected into a blood vessel or into the cerebrospinal fluid. The latter complication can be avoided by not advancing the needle more than 24 mm from the entry site and by asking the patient to look straight ahead (as proved by CT scan studies). Systemic complications manifest as circulatory collapse, disturbance in the level of consciousness (drowsiness), pulse irregularities, or convulsions.
